# Support for the wellbeing of frontline healthcare workers should be incorporated in health emergency preparedness planning

**DOI:** 10.1038/s43856-025-01367-8

**Published:** 2026-01-16

**Authors:** Kate McNeil, Stephanie Nzekwu, Lucy Gilson, Alex Hinga, Dorothy Oluoch, Yingxi Zhao, Sassy Molyneux

**Affiliations:** 1 Health Systems Collaborative, Nuffield Department of Medicine, University of Oxford, Oxford, United Kingdom; 2 Pandemic Sciences Institute, University of Oxford, Oxford, United Kingdom; 3 Health Policy and Systems Division, School of Public Health, University of Cape Town, Cape Town, South Africa; 4 Department of Global Health and Development, London School of Hygiene and Tropical Medicine, London, United Kingdom; 5 KEMRI-Wellcome Trust Research Programme, Kilifi, Kenya; 6 KEMRI-Wellcome Trust Research Programme, Nairobi, Kenya

**Keywords:** Health services, Public health

## Abstract

Health system resilience requires a resilient and well workforce. During health crises, including pandemics and outbreaks of pathogens, frontline healthcare workers can face significant challenges, resulting in personal psychosocial costs for staff members, and organisational consequences for the health systems they work within. Here, we highlight that there is an urgent need to proactively incorporate organisational measures to protect and promote the wellbeing of frontline healthcare staff across all stages of response to health crises. Importantly, this not only involves specific emergency preparedness and planning efforts, but also supporting organisational-level everyday practices that foster staff wellbeing and health system resilience outside of crises.

## Abstract (web summary)

McNeil et al. discuss the need to proactively protect and promote the wellbeing of frontline healthcare staff within the everyday practices of health organizations and in health emergency planning and response. They highlight how health crises add to the routine stresses facing health systems, explore challenges facing staff, and suggest next steps and potential solutions as well as the potential risks of inaction

## Introduction

More than five years on from the onset of the COVID-19 pandemic, health systems are continuing to experience the consequences of the health crisis on their workforces, including an increase in healthcare staff turnover and retention issues^1^, significant post-pandemic levels of job dissatisfaction and burnout^2^, and having to integrate new practices and narratives informed by crisis experiences^3^. A growing body of research is examining the impact of the COVID-19 pandemic on healthcare worker wellbeing, the coping strategies adopted by healthcare workers, and the steps taken to support healthcare worker wellbeing during the crisis, including work focused on the wellbeing of nurses^4^, and early career doctors^5^. Staff support strategies that act at both the individual and organisational levels should be integral to health system responses throughout all phases of health emergency responses, including preparedness and response phases. Moreover, supporting workforce wellbeing and organisational resilience within the routine everyday practices of health systems can contribute to developing systems which are more resilient to shocks and crisis events when they do happen.

In this Perspective, we describe some of the risks to staff wellbeing during health crises; identify gaps in research and policy in responding to these risks; explore potential consequences of insufficient support for the prevention of psychological harm before, during, and after health emergencies; and identify areas in which researchers, health systems leaders, and governance actors might take additional steps to address the needs of healthcare workers in preparedness for and responses to health emergency events. We use literature from our various research and practice backgrounds in a range of High-Income and Low- and Middle-Income Country (LMIC) health systems contexts, including work in health policy and systems research, organisational management, public policy, ethics, and public health. Throughout, we draw on the COVID-19 pandemic as a recent health crisis example and relevant source of potential learning.

We focus primarily on organisational interventions and measures to support resilience and wellbeing, which we define as interventions focusing proactively on the workplace or working conditions^6^. While individual-level initiatives such as mindfulness-based training or mobile health applications may have some potential promise in supporting mental health during crises^7^, these initiatives also risk adding to individual burdens and protecting only those workers who engage with them^6^. Organisational measures that protect the wider workforce are therefore essential.

For definitional and conceptual clarity, we define resilience as the capability to cope with or adapt to chronic or acute stressors, including shocks at the individual or organisational level^8,9^. We place particular emphasis on organisational resilience as a potential facilitator of workplace environments that enable space for individual resilience and wellbeing, while stressing that there are limits to the resilience that can be expected of individuals during crisis events. Wellbeing is a multi-faceted positive state which supports human flourishing^9^. Emotional distress as a challenge to wellbeing may take a variety of forms, including moral injury, moral distress, post-traumatic stress, and anxiety.

### Risks to staff wellbeing during health crises

Frontline healthcare workers are a diverse group of medical, nursing, and allied health professionals who have direct contact with patients in the regular course of their duties in facilities or communities. They face a range of challenges to their resilience during health crises. They are vulnerable to morally injurious events triggered by limits to conventional professional knowledge and values, or the ability to apply these^10,11^. Also, their expertise can be challenged in situations where there is uncertainty about best practices^11^. Patient-facing healthcare workers can be exposed to hypervigilance and multiple forms of prolonged uncertainty within the workplace^12^, heavy workloads with insufficient personal protective equipment (PPE)^13^, stigmatisation within their local communities^14^, and risks to their physical health^15,16^. The multiple challenges for frontline healthcare workers associated with a health crisis pose significant risks for the mental health and wellbeing of frontline healthcare workers^17–19^, with findings from the COVID-19 pandemic highlighting risks such as post-traumatic stress symptoms, burnout^20^ professional grief^21^, moral distress and compassion fatigue^21^.

Challenges for frontline staff have the potential to become widespread during a health crisis. Rates of burnout during the COVID-19 pandemic varied both regionally and over time: one international meta-analysis focused on physicians found a burnout prevalence of 60.7% early in the pandemic^22^, while an umbrella review and meta-analysis of meta-analyses has suggested that there was a prevalence ratio of 0.44 of burnout symptoms and 0.32 of psychological distress for healthcare workers across the COVID-19 pandemic^23^. An umbrella review of meta-analyses focused on the prevalence of anxiety and depression in healthcare workers during the pandemic estimated a prevalence of 24.94% for healthcare worker anxiety globally and 24.83% for healthcare worker depression during the COVID-19 pandemic^24^. While these numbers may be limited in their ability to reveal rich nuance and variation within experiences of the COVID-19 pandemic, they suggest that emotional challenges were pervasive. Challenges to wellbeing may have been entangled with manifestations of moral distress linked to experiences of “moral events”^25^, commonly defined as circumstances where staff do not know what the right thing to do is, or do know, but are unable to take that course of action as a result of organisational constraints^25^. Moral distress can be experienced as moral injury, which has been defined as “where sustained moral distress leads to impaired function or longer-term psychological harm”^26^.

Some influenza plans and pandemic preparedness planning documents recognise the burden placed upon healthcare workforces during these crisis events. For example, there is recognition that “many staff will have been working under acute pressure for prolonged periods” in the United Kingdom’s Influenza Plan^27^; and recognition of a need to “address the psychological impacts of the pandemic, especially on the health workforce”^28^. However, insufficient attention has been paid to what might be done routinely, and as part of crisis preparedness.

### Health crises add to routine stresses and challenges for health systems

Crisis events such as the COVID-19 pandemic can act as an additional shock to individuals and the health systems in which they work, adding to the routine stresses and everyday challenges of providing health care^29^. Such additional shocks to the health system have the potential to act as amplifying events, making frontline healthcare staff more vulnerable to some of the detrimental impacts and risks posed by a trauma-heavy work environment at the moment when the health system is under great pressure and needs them most^30^. This means that pre-existing challenges faced by individuals and health systems may be exacerbated by crises. There is evidence that healthcare workers face an array of work-related challenges to their wellbeing under normal operating conditions that health systems leaders must address. This requires engaging the relational and organisational influences on individual resilience, such as staffing levels, design and implementation of institutional policies, nature of teamwork and leadership style^29,31,32^. Inadequate staffing levels, challenging inter-personal relationships within teams, inadequate resources, high mortality rates and hierarchical leadership styles can expose health workers to adverse psychological and emotional harm including moral frustrations, moral distress and mental health conditions as they struggle to reconcile their expectations with the realities of practice^33,34^.

In the course of performing their normal daily roles, healthcare workers can therefore face poor working environments or be exposed to disruptions and traumatic events without adequate preparation and support. They can face challenges to their physical safety and to their emotional wellbeing. Uncertainty, change, and chronic stressors within the health system can challenge the everyday resilience of staff and the organisations they work for while organisational practices, relationships, and networks can support and facilitate this resilience^29^. In ordinary operations, and in preparing for and responding to health emergencies, awareness of and responsiveness to these sources of, and barriers to, resilience is essential.

### The risks of inaction

Health systems have an ethical duty of care to those they employ and to those depending on the system for the delivery of quality care. Supporting organisations in their efforts to meet this duty and to take reasonable steps to prevent their workforce from experiencing forms of psychological harm warrants systems-level action. This includes incorporating workforce wellbeing support into pandemic preparedness efforts. Alongside this ethical duty of care, organisations face a range of further business-case motivations to invest in employee wellbeing, including legal and regulatory obligations, fostering a positive organisational reputation, and motivating staff to perform well^35^. Work conducted outside of health emergencies has also suggested that workforce stability may contribute to productivity^36^.

Poor healthcare workforce wellbeing poses risks not just for healthcare workers themselves, but also for health systems and the patients and communities they serve. Burnout and poor wellbeing amongst healthcare staff have been linked to poorer patient safety outcomes^37^; including during the COVID-19 pandemic, as a result of staff competencies, quality of care and professional standards being undermined^38^. Such impacts can endure in the aftermath of a health crisis, with one descriptive study from Thailand demonstrating a relationship between burnout in nurses and missed nursing care after the COVID-19 pandemic^39^. Poor staff wellbeing can also contribute to problems with retaining staff, resulting in personnel shortages, loss of vital institutional memory, and reduced access to experienced staff members during emergencies^40^. Staff perceptions of organisational injustice have been associated with departure and withdrawal^41,42^. Poor treatment in the workplace and inadequate access to the resources needed to uphold the values of care delivery challenge organisational justice and trust across a health system, in both normal times and health crises.

The values of a health profession are judged by its’ practices, and so healthcare workers need to be seen to act in the interests of the public and wider society. These values link to the interests of professionals themselves who seek to maintain their trustworthiness, legitimacy and mandate^43–45^. Patients are not the only ones who suffer when expected ideals are not upheld. For health workers, extended partaking in or witnessing of continuous degrading of core professional values by external conditions can challenge wellbeing and cause moral injury^46,47^. Moral injury can foster deep emotional wounds that may be resistant to mechanisms for self-forgiveness, self-compassion and survival. In professional work, this can be counterproductive. For example, studies in high-income countries (e.g. refs. ^48^,^49^) suggest that heightened and unattended moral injury during the COVID-19 pandemic lead to impaired performance of health workers and negative patient outcomes. Meanwhile, work conducted prior to the COVID-19 pandemic has suggested that the moral climate can also be negatively affected. For example, in a setting where there were frequent moral transgressions and disappointments some nurses abandoning trying to work in line with moral codes^50^.

The consequences for downplaying preparedness for health emergencies are far-reaching, and health systems conditions need to be created that allow frontline staff to meet the standard of care while protecting their well-being in all times.

### Implementing next steps and potential solutions

Throughout health crises, including pandemics and infectious disease outbreaks of priority pathogens, the capacity and resilience of responding health systems can only be as resilient as the individuals working at the frontline of those systems. A range of actors within research communities, health systems, and policy have a role to play in ensuring that the appropriate steps are taken to protect the psychosocial wellbeing of frontline healthcare workers in their work on pandemic preparedness.

#### Undertaking research

Health systems researchers should continue to deepen their understanding of many inter-related barriers and facilitators of psychological wellbeing and resilience amongst frontline healthcare staff in routine practice and before, during, and after health emergencies. We suggest the following foci for research:

While there is a growing body of evidence highlighting the harms faced by healthcare workers during health emergency events; the evidence base concerning interventions to address this challenge is weaker^51–53^, with one rapid review of reviews noting a “glaring lack”^54^ of recent academic evidence. We believe that work is needed to identify context relevant interventions and strategies that can act at the organisational level. Here, there is potential to learn from the experiences of other trauma-affected frontline sectors, including insights from humanitarian workforces and those operating in disaster and conflict response settings. This might include adapting learning from the practice of Schwartz Rounds in routine healthcare settings, and vicarious trauma training and Hostile Environment Awareness Training in humanitarian contexts^55^, health system future-proofing initiatives^56^, and evaluations of staff support protocols and training programmes^57^.

In a field currently dominated by work emerging from the COVID-19 pandemic, there is a need to deepen our understanding of challenges and interventions across a greater array of crisis events. It is essential that voices, practices, and experiences from a range of cultural and resource contexts, including those that have experienced different health emergency events are well-represented in this work.

Where there is policy guidance aimed at supporting frontline staff, it often places an emphasis on supporting staff in their recovery from trauma during the recovery phase following a health emergency, instead of incorporating the prevention of psychological harm into routine working practices and into the early onset stages of response. We should increase our understanding of how work on everyday resilience within health systems might be applied to our understanding of preparedness for, and responses to, shock events.

Some categories of staff are under-represented within literature on organisational interventions to support staff during health emergencies, including frontline research staff, students, community health workers and trainees^58^. While some valuable work has documented interventions to support community healthcare workers in LMIC settings during the COVID-19 pandemic^59^, such groups need to be actively incorporated into future work. Work on organisational-level interventions within health systems could draw insights from routine practices and health emergencies, and should be conducted in collaboration with health systems actors from diverse cultural and resource contexts. The aim would be to develop an evidence base to inform interventions and practices which are feasible, affordable, and culturally appropriate in the short and long-term.

All of the above work could incorporate insights from health system resilience work (e.g. refs. ^29^,^31^). Work on everyday health system resilience provides learning on a variety of challenges to resilience^31^, as well as on absorptive, adaptive, and transformative strategies with the potential to protect and build resilience^60^. Some researchers emphasise the importance of taking into account considerations of power and relational dynamics^31^, encompassing hierarchical authority, reward provision, task allocation and division, and role clarity. Sensitivity to formal and informal dynamics can reduce potential conflict and encourage collaboration during times of uncertainty.

#### Changing policy and practice

Health systems leaders should seek to integrate known best practices for supporting staff wellbeing into routine organisational practices and pandemic preparedness. They need support to embed practices which reduce the background risk of psychological harm posed by everyday systems functioning challenges, and to develop evidence-informed guidance to support additional measures appropriate to every stage of a health emergency response. Potentially useful practices include provision of access to peer support groups^61^ and reflective debriefing sessions^62^.

Both during and after emergency events, health systems leaders and stakeholders should look for opportunities to identify local learning surrounding what worked, and to share their insights into best practices. Sharing their learning with colleagues, partners, and academic communities might support the translation of best practices into ‘new normals’ that strengthen preparedness for future health crises. Initiatives at the organisational level need national and international level support. At the national level, governments should ensure pandemic response policies and plans include proactive efforts to protect frontline healthcare staff from psychological harm across every phase of a pandemic response, and support system resilience. Learning from the Nuffield global health emergency research ethics guidance^63^, this might include capacity strengthening initiatives, ensuring that allocation of duties and organisational practices are respectful and fair. Support for leaders and managers who may also face emotional distress and other challenges during health crises^64^ and in their day-to-day work^65^, is also likely to be needed. At the international level, WHO influenza planning incorporates the planned provision of psychological support for healthcare workers at the widespread infection stage^28^. This approach should be expanded to explore how minimising the risk of psychological harm to healthcare workers might also be incorporated into earlier stages of outbreak response.

### Concluding remarks

Leadership across health systems organisations, research institutions, and multiple levels of policymaking have roles to play in identifying, developing, implementing, and iterating efforts to protect and promote frontline healthcare worker wellbeing in health emergency preparedness planning and in the everyday functioning of health organisations. The COVID-19 pandemic brought the challenges facing staff wellbeing – and the potential risks of inaction for individuals and our health systems – into the foreground. This window of opportunity for learning and action should be used as a foundation from which to build supportive practices fostering individual wellbeing and organisational resilience into institutional planning and operational practice both within and outside of health crises.

## References

[1] Shen, K., Eddelbuettel, J. C. P. & Eisenberg, M. D. Job flows into and out of health care before and after the COVID-19 pandemic. *JAMA Health Forum* **5**, e234964 (2024). 38277171 10.1001/jamahealthforum.2023.4964 PMC10818214

[2] Galanis, P. et al. Increased job burnout and reduced job satisfaction for nurses compared to other healthcare workers after the COVID-19 pandemic. *Nurs. Rep.* **13**, 1090–1100 (2023). 37606463 10.3390/nursrep13030095 PMC10443294

[3] Kent, D. & Dacin, M. T. After the crisis: explaining stories of professional identity growth from collective action. *Organ. Stud*. 01708406241295495, 10.1177/01708406241295495 (2024). 10.1177/01708406241295495 PMC12182274 40547512

[4] Browne, C. & Chun Tie, Y. Promoting well-being: a scoping review of strategies implemented during the COVID-19 pandemic to enhance the well-being of the nursing workforce. *Int. J. Nurs. Stud. Adv.* **6**, 100177 (2024). 38746802 10.1016/j.ijnsa.2024.100177 PMC11080544

[5] Graham, A. & Dale, A.-M. Wellbeing support for foundation doctors during COVID-19 in GHNHSFT. *BJPsych Open* **7**, S189 (2021).

[6] Fox, K. E. et al. Organisational- and group-level workplace interventions and their effect on multiple domains of worker well-being: a systematic review. *Work Stress* **36**, 30–59 (2022).

[7] Ezeonu, N. A. et al. Mobile apps to support mental health response in natural disasters: scoping review. *J. Med. Internet Res.* **26**, e49929 (2024). 38520699 10.2196/49929 PMC11063879

[8] Gilson, L. et al. Everyday resilience in district health systems: emerging insights from the front lines in Kenya and South Africa. *BMJ Glob. Health* **2**, e000224 (2017). This article describes how frontline leaders in health systems cope with the multiple concurrent crises and everyday stressors they face within their institutions. This article has influenced our thinking around the relationships between health crisis responses, routine practices and system resilience-building. 29081995 10.1136/bmjgh-2016-000224 PMC5656138

[9] Sayed, T., Malan, H. & Fourie, E. Exploring the associations between resilience and psychological well-being among South Africans during COVID-19. *Front. Psychol*. **15**, 1323466 (2024). 10.3389/fpsyg.2024.1323466 PMC10898365 38414871

[10] Hegarty, S. et al. It hurts your heart’: frontline healthcare worker experiences of moral injury during the COVID-19 pandemic. *Eur. J. Psychotraumatol.* **13**, 2128028 (2022). 36276556 10.1080/20008066.2022.2128028 PMC9586685

[11] Compagni, A., Cappellaro, G. & Nigam, A. Responding to professional knowledge disruptions of unmitigable uncertainty: the role of emotions, practices, and moral duty among COVID-19 physicians. *Acad. Manage. J*. 10.5465/amj.2022.0697 (2023).

[12] Berkhout, S. G. et al. Shared sources and mechanisms of healthcare worker distress in COVID-19: a comparative qualitative study in Canada and the UK. *Eur. J. Psychotraumatol.* **13**, 2107810 (2022). 35979505 10.1080/20008066.2022.2107810 PMC9377263

[13] Oyat, F. W. D. et al. The psychological impact, risk factors and coping strategies to COVID-19 pandemic on healthcare workers in the sub-Saharan Africa: a narrative review of existing literature. *BMC Psychol.* **10**, 284 (2022). 36457038 10.1186/s40359-022-00998-z PMC9714392

[14] McMahon, S. A. et al. Healthcare providers on the frontlines: a qualitative investigation of the social and emotional impact of delivering health services during Sierra Leone’s Ebola epidemic. *Health Policy Plan* **31**, 1232–1239 (2016). 27277598 10.1093/heapol/czw055 PMC5035780

[15] Matz, M. et al. Excess mortality among essential workers in England and Wales during the COVID-19 pandemic. *J Epidemiol. Community Health* **76**, 660–666 (2022). 35470261 10.1136/jech-2022-218786

[16] Shah, A. S. V. et al. Risk of hospital admission with coronavirus disease 2019 in healthcare workers and their households: nationwide linkage cohort study. *BMJ* **371**, m3582 (2020). 33115726 10.1136/bmj.m3582 PMC7591828

[17] Johnson, S. U., Ebrahimi, O. V. & Hoffart, A. PTSD symptoms among health workers and public service providers during the COVID-19 outbreak. *PLoS One* **15**, e0241032 (2020). 33085716 10.1371/journal.pone.0241032 PMC7577493

[18] Lu, M.-Y. et al. The prevalence of post-traumatic stress disorder symptoms, sleep problems, and psychological distress among COVID-19 frontline healthcare workers in Taiwan. *Front. Psychiatry* **12**, 705657 (2021). 10.3389/fpsyt.2021.705657 PMC8312888 34322044

[19] Dawood, B., Tomita, A. & Ramlall, S. Unheard,’ ‘uncared for’ and ‘unsupported’: the mental health impact of Covid −19 on healthcare workers in KwaZulu-Natal Province, South Africa. *PLOS ONE* **17**, e0266008 (2022). 35507540 10.1371/journal.pone.0266008 PMC9067674

[20] Aymerich, C. et al. COVID-19 pandemic effects on health worker’s mental health: systematic review and meta-analysis. *Eur. Psychiatry* **65**, e10 (2022). 35060458 10.1192/j.eurpsy.2022.1 PMC8828390

[21] Rabow, M. W., Huang, C.-H. S., White-Hammond, G. E. & Tucker, R. O. Witnesses and victims both: healthcare workers and grief in the time of COVID-19. *J. Pain Symptom Manage.* **62**, 647–656 (2021). 33556494 10.1016/j.jpainsymman.2021.01.139 PMC7864782

[22] Macaron, M. M. et al. A systematic review and meta analysis on burnout in physicians during the COVID-19 pandemic: a hidden healthcare crisis. *Front. Psychiatry* **13**, 1397 (2023). 10.3389/fpsyt.2022.1071397 PMC9877514 36713915

[23] Boucher, V. G. et al. An umbrella review and meta-analysis of 87 meta-analyses examining healthcare workers’ mental health during the COVID-19 pandemic. *J. Affect. Disord.* **375**, 423–436 (2025). 39862981 10.1016/j.jad.2025.01.109

[24] Sahebi, A. et al. The prevalence of anxiety and depression among healthcare workers during the COVID-19 pandemic: an umbrella review of meta-analyses. *Prog. Neuropsychopharmacol. Biol. Psychiatry* **107**, 110247 (2021). 33476692 10.1016/j.pnpbp.2021.110247 PMC7817526

[25] Morley, Ives, G., Bradbury-Jones, J. & Irvine, C. F. What is ‘moral distress’? A narrative synthesis of the literature. *Nurs. Ethics* **26**, 646–662 (2019). 28990446 10.1177/0969733017724354 PMC6506903

[26] British Medical Association. *Moral Distress and Moral Injury: Recognising and Tackling it for UK Doctors* (British Medical Association, 2021).

[27] Department of Health. *UK Pandemic Influenza Preparedness Strategy**2011*. https://assets.publishing.service.gov.uk/media/5a7c4767e5274a2041cf2ee3/dh_131040.pdf (2011).

[28] WHO Global Influenza Programme & World Health Organization. *Pandemic Influenza Preparedness and Response: A WHO Guidance Document*, 60 (WHO, 2009). 23741778

[29] Gilson, L. et al. Everyday resilience in district health systems: emerging insights from the front lines in Kenya and South Africa. *BMJ Glob. Health* **2**, e000224 (2017). 10.1136/bmjgh-2016-000224 PMC5656138 29081995

[30] Molinaro, M. L. Moral distress: a structural problem with individual solutions. *J. Health Serv. Res. Policy* **30**, 77–78 (2025). 39851218 10.1177/13558196251315330

[31] Witter, S. et al. Health system resilience: a critical review and reconceptualisation. *Lancet Glob. Health* **11**, e1454–e1458 (2023). 37591591 10.1016/S2214-109X(23)00279-6

[32] Gilson, L., Ellokor, S., Lehmann, U. & Brady, L. Organizational change and everyday health system resilience: Lessons from Cape Town, South Africa. *Soc. Sci. Med.* **266**, 113407 (2020). 33068870 10.1016/j.socscimed.2020.113407 PMC7538378

[33] Heaphy, E. D. Repairing breaches with rules: maintaining institutions in the face of everyday disruptions. *Organ. Sci.* **24**, 1291–1315 (2013).

[34] Wright, A. L., Zammuto, R. F. & Liesch, P. W. Maintaining the values of a profession: institutional work and moral emotions in the emergency department. *Acad. Manage. J.* **60**, 200–237 (2017).

[35] Henstock, L., Johnson, R., Kinghorn, P., Beach, D. & Al-Janabi, H. Why and how do workplaces invest in mental health and wellbeing? A systematic review and process tracing study. *Soc. Sci. Med.* **366**, 117633 (2025). 39721167 10.1016/j.socscimed.2024.117633

[36] Buchan, J. Reviewing the benefits of health workforce stability. *Hum. Resour. Health* **8**, 29 (2010). 21156050 10.1186/1478-4491-8-29 PMC3016248

[37] Hall, L. H., Johnson, J., Watt, I., Tsipa, A. & O’Connor, D. B. Healthcare staff wellbeing, burnout, and patient safety: a systematic review. *PLOS One* **11**, e0159015 (2016). 27391946 10.1371/journal.pone.0159015 PMC4938539

[38] Mogomotsi, G. & Creese, J. European nurses’ burnout before and during the COVID-19 pandemic and its impact on patient safety: a scoping review. *Hospitals* **1**, 151–171 (2024).

[39] Nantsupawat, A., Wichaikhum, O.-A., Abhicharttibutra, K., Sadarangani, T. & Poghosyan, L. The relationship between nurse burnout, missed nursing care, and care quality following COVID-19 pandemic. *J. Clin. Nurs.* **32**, 5076–5083 (2023). 37219019 10.1111/jocn.16761

[40] Martin, C. A. et al. Factors associated with attrition from the UK healthcare workforce since the COVID-19 pandemic: results from a nationwide survey study. *Lancet Reg. Health Eur*. **47**, 101133 (2024). 10.1016/j.lanepe.2024.101133 PMC11670675 39726719

[41] Turnley, W. H. & Feldman, D. C. The impact of psychological contract violations on exit, voice, loyalty, and neglect. *Hum. Relat.* **52**, 895–922 (1999).

[42] Baldwin, S. *Organisational Justice* (Institute for Employment Studies Brighton, 2006).

[43] Abbott, A. *The System of Professions: An Essay on the Division of Expert Labor*. xvi, 435 (The University of Chicago Press, 1988).

[44] Professionalism: Value and ideology - Julia Evetts. 10.1177/0011392113479316 (2013).

[45] Goode, W. J. Community within a community: the professions. *Am. Sociol. Rev.* **22**, 194–200 (1957).

[46] Litz, B. T. et al. Moral injury and moral repair in war veterans: a preliminary model and intervention strategy. *Clin. Psychol. Rev.* **29**, 695–706 (2009). 19683376 10.1016/j.cpr.2009.07.003

[47] Dean, W., Talbot, S. & Dean, A. Reframing clinician distress: moral injury not burnout. *Fed. Pract.* **36**, 400–402 (2019). 31571807 PMC6752815

[48] Dean, W., Jacobs, B. & Manfredi, R. A. Moral injury: the invisible epidemic in COVID health care workers. *Ann. Emerg. Med.* **76**, 385–386 (2020). 32586634 10.1016/j.annemergmed.2020.05.023 PMC7308739

[49] Hossain, F. & Clatty, A. Self-care strategies in response to nurses’ moral injury during COVID-19 pandemic. *Nurs. Ethics* **28**, 23–32 (2021). 33124492 10.1177/0969733020961825 PMC7604672

[50] Hakimi, H., Joolaee, S., Ashghali Farahani, M., Rodney, P. & Ranjbar, H. Moral neutralization: Nurses’ evolution in unethical climate workplaces. *BMC Med. Ethics* **21**, 1–10 (2020). 33203415 10.1186/s12910-020-00558-3 PMC7672869

[51] Nicolakakis, N. et al. Are organizational interventions effective in protecting healthcare worker mental health during epidemics/pandemics? A systematic literature review. *Int. J. Environ. Res. Public. Health* **19**, 9653 (2022). 35955009 10.3390/ijerph19159653 PMC9368524

[52] Belita, E. et al. Organizational interventions to support and promote the mental health of healthcare workers during pandemics and epidemics: a systematic review. *BMC Health Serv. Res.* **25**, 731 (2025). 40394626 10.1186/s12913-025-12888-2 PMC12093622

[53] Pollock, A. et al. Interventions to support the resilience and mental health of frontline health and social care professionals during and after a disease outbreak, epidemic or pandemic: a mixed methods systematic review. *Cochrane Libr*. 10.1002/14651858.CD013779/full (2020). This systematic review discusses the state of the evidence base surrounding interventions to support the wellbeing of frontline health staff during health emergencies, up until the early stages of the covid-19 pandemic. This piece highlights some of the gaps in the evidence base surrounding interventions to support frontline staff during health crises, which we argue needs to be addressed going forward. 10.1002/14651858.CD013779 PMC8226433 33150970

[54] Frias, C. E. et al. Strategies to support the mental health and well-being of health and care workforce: a rapid review of reviews. *Front. Med*. **12**, 1530287 (2025). 10.3389/fmed.2025.1530287 PMC11961965 40177285

[55] Headington Institute. Global aid. Disaster relief. Community response. https://headington-institute.org/ (2025).

[56] The Black Dog Institute. *Future Proofing the Frontline: Organisational Strategies to Support the Mental Health and Wellbeing of Healthcare Workers during Crises* (The Black Dog Institute, 2024).

[57] Abdul Rahim HF, Fendt-Newlin M, Al-Harahsheh ST, Campbell J. Our duty of care: A global call to action to protect the mental health of health and care workers. Doha, Qatar: World Innovation Summit for Health, (2022).

[58] Klasen, J. M. et al. Medical students on the COVID-19 frontline: a qualitative investigation of experiences of relief, stress, and mental health. *Front. Med*. **10**, 1249618 (2023). 10.3389/fmed.2023.1249618 PMC10666052 38020159

[59] Yakubu, K. et al. An intervention package for supporting the mental well-being of community health workers in low, and middle-income countries during the COVID-19 pandemic. *Compr. Psychiatry* **115**, 152300 (2022). 35276492 10.1016/j.comppsych.2022.152300 PMC8881902

[60] Gilson, L., Barasa, E., Cloete, K., Kasera, K., Tsofa, B. & Vallabhjee, K. Chapter 24: What can we learn about everyday health system resilience and pandemic response and preparedness from Kenyan and South African COVID-19 experiences? In Handbook of Health System Resilience. Cheltenham, UK: Edward Elgar Publishing. 10.4337/9781803925936.00036 (2024).

[61] Peterson, U., Bergström, G., Samuelsson, M., Åsberg, M. & Nygren, Å Reflecting peer-support groups in the prevention of stress and burnout: randomized controlled trial. *J. Adv. Nurs.* **63**, 506–516 (2008). 18727753 10.1111/j.1365-2648.2008.04743.x

[62] Browning, E. D. & Cruz, J. S. Reflective debriefing: a social work intervention addressing moral distress among ICU nurses. *J. Soc. Work End Life Palliat. Care* **14**, 44–72 (2018). 29488856 10.1080/15524256.2018.1437588

[63] Nuffield Council on Bioethics. *Research in Global Health Emergencies: Ethical Issues*. https://www.nuffieldbioethics.org/publication/research-in-global-health-emergencies-ethical-issues/. This report explores research ethics in health emergencies, including, in Chapter 10, describing challenges facing frontline researchers during global health emergencies and providing accompanying recommendations. In our Perspectives piece, we cite the Nuffield Council on Bioethics guidance as an example of the type of materials we believe might be useful to see developed within the frontline healthcare staff space (2020).

[64] Udod, S., Baxter, P., Gagnon, S., Halas, G. & Raja, S. Experiences of frontline managers during the COVID-19 pandemic: recommendations for organizational resilience. *Healthcare* **12**, 407 (2024). 38338292 10.3390/healthcare12030407 PMC10855724

[65] Hertelendy, A. J. et al. Mitigating moral distress in leaders of healthcare organizations: a scoping review. *J. Healthc. Manag. Am. Coll. Healthc. Exec*. **67**, 380–402 (2022). 10.1097/JHM-D-21-00263 36074701

